# Data management for prospective research studies using SAS^® ^software

**DOI:** 10.1186/1471-2288-8-61

**Published:** 2008-09-11

**Authors:** Robin L Kruse, David R Mehr

**Affiliations:** 1 Department of Family and Community Medicine, University of Missouri School of Medicine, Columbia, MO, 65212, USA

## Abstract

**Background:**

Maintaining data quality and integrity is important for research studies involving prospective data collection. Data must be entered, erroneous or missing data must be identified and corrected if possible, and an audit trail created.

**Methods:**

Using as an example a large prospective study, the Missouri Lower Respiratory Infection (LRI) Project, we present an approach to data management predominantly using SAS software. The Missouri LRI Project was a prospective cohort study of nursing home residents who developed an LRI. Subjects were enrolled, data collected, and follow-ups occurred for over three years. Data were collected on twenty different forms. Forms were inspected visually and sent off-site for data entry. SAS software was used to read the entered data files, check for potential errors, apply corrections to data sets, and combine batches into analytic data sets. The data management procedures are described.

**Results:**

Study data collection resulted in over 20,000 completed forms. Data management was successful, resulting in clean, internally consistent data sets for analysis. The amount of time required for data management was substantially underestimated.

**Conclusion:**

Data management for prospective studies should be planned well in advance of data collection. An ongoing process with data entered and checked as they become available allows timely recovery of errors and missing data.

## Background

Data that are highly reliable and complete are essential to unbiased, high-quality research studies [1,2]. While poor statistical analyses can be run again, "...a badly designed study with inferior data is beyond the redemption of the most sophisticated statistical technique" [3]. Prospective data collection gives researchers control over the quality of their data. Mistakes and omissions are likely to occur, however, regardless of how well-designed the study and how careful the study personnel [1,2]. Thus, it is essential that researchers develop and implement procedures to minimize data loss, identify concerns soon after data are collected, and detect and correct errors [1,2,4,5]. "No study is better than the quality of the data" [6].

If detection of data errors is delayed, they become more difficult to correct [4,6,7]. Possible sources of error occur throughout a study, and include deviations from the study protocol; inaccurate equipment; poorly designed forms; illegible, inaccurate, or incomplete data recording; errors or omissions in data transfer; inadequate training; intentional fraud; undocumented changes; programming errors; and misuse of statistical software [8]. It is therefore essential that data quality control–the detection, review and correction of errors in data that have been collected–begins in the design stage of prospective studies and continues throughout data collection [2,5-8]. The ability to give regular and rapid feedback to investigators and data collectors highlights preventable problems in the data collection process and prevents deterioration in data quality [1,9].

In this paper we present the data management system used in a large prospective project conducted at two centers. The principles that guided our process could apply to virtually any project. While there are now sophisticated systems available for data management, most are quite expensive. Pilot studies and preliminary investigations are often unfunded, and must rely on existing resources.

## Methods

### Study setting

The Missouri Lower Respiratory Infection (LRI) Project was a large prospective cohort study of outcomes (mortality and functional change) of nursing home residents who developed an LRI [10,11]. The protocol was approved by institutional review boards at two medical centers, several independent hospitals, and two nursing home ethical review panels. Conducted in central Missouri and the St. Louis area, the study enrolled subjects from August 1995 through September 1998, and data collection continued for an additional three months.

Our institutional review board helped develop an appropriate strategy for enrolling participants. We contacted attending physicians in all facilities that had agreed to participate in the study. Physicians either declined to participate, or agreed to have trained study nurses provide timely, comprehensive evaluations of their residents who developed an illness consistent with an LRI. Physicians could *a priori *exclude any resident from evaluation. The study nurse recorded initial data and quickly communicated findings to the resident's physician, usually by facsimile transmission. Treatment decisions were left to the attending physician. Because these detailed evaluations were authorized by attending physicians who received clinical information and made treatment decisions accordingly, evaluations were considered part of appropriate care. For this reason, institutional review boards allowed a simplified consent process consisting of a simple refusal or acceptance of the clinical evaluation by the resident or a family member.

Study enrollment was a two-step process [10]. Criteria for evaluating, excluding, and enrolling residents are shown in Table 1. First, after eliminating residents with exclusion reasons, eligible patients with illness signs and symptoms compatible with an LRI were evaluated. Based on the evaluation and chest radiograph results, residents who met the LRI definition (Table 1) were enrolled. We refer the reader to Mehr et al. [10] for further details regarding evaluation and enrollment. Residents could be enrolled multiple times, providing that they were well and off antibiotics for at least seven days following the previous episode. In the analysis, we used general estimating equations to adjust for individuals being represented in the data more than once.

**Table 1:** Criteria for evaluating, excluding, and enrolling residents in the Missouri LRI Project.

Residents eligible for evaluation met one or more of the following three criteria:
1. Two or more new lower respiratory symptoms (e.g., cough, shortness of breath, cyanosis)
2. One new respiratory symptom and at least one sign of an acute change in condition (e.g., fever, decreased alertness, new or increased confusion)
3. At least one sign of an acute change in condition and no evidence of stroke, gastroenteritis, urine infection, constipation/fecal impaction, or an adverse drug reaction
Residents were excluded from evaluation if they met one or more of the following criteria:
1. Did not meet evaluation criteria (above)
2. Resident or a family member declined evaluation, or resident's physician excluded them from the protocol
3. Resident's physician was not signed on to the protocol
4. Resident was not well and off antibiotics for at least seven days following a prior LRI
5. Resident was not at least 60 years of age
6. Resident had less than one month life expectancy, resident was a hospice patient, or resident had AIDS
7. Resident had a "no antibiotics" order in effect
8. Illness episode was missed
9. Resident had not been in facility for at least 14 days
The six enrollment criteria were
1. New or increased cough
2. New or increased sputum production
3. Fever
4. Pleuritic chest pain
5. New or increased physical findings on chest examination (rales, rhonchi, wheezes, or bronchial breathing)
6. One or more indications of change in status or breathing difficulty (new or increased shortness of breath, respiratory rate > 25, and worsening mental or functional status)
Residents were enrolled if, after evaluation, they met three or more of the above enrollment criteria, or they met two criteria and had chest x-ray findings positive for pneumonia. We further required that residents with congestive heart failure or chronic obstructive pulmonary disease had either a fever or a chest x-ray that was positive for pneumonia to avoid confusing an acute exacerbation of their condition with an LRI.

**Figure 1:**
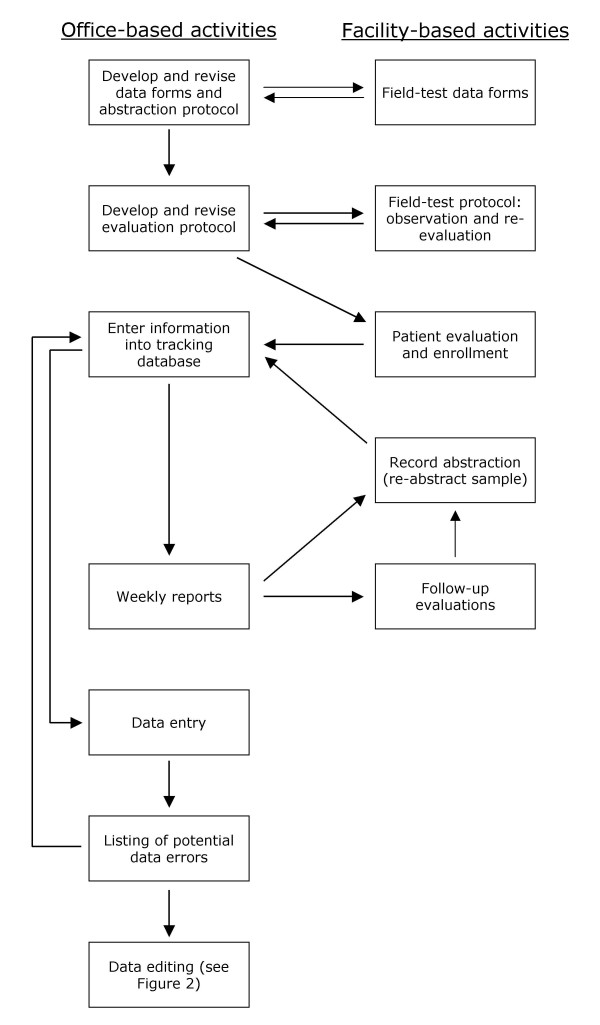
Flowchart of organizational tasks (Note: some tasks such as obtaining IRB approval, obtaining facility participation, and interacting with attending physicians are not included).

Evaluation information was subsequently abstracted from medical records without recording personal identifiers on the abstraction forms. Other data were obtained by medical record abstraction and follow-up visits with surviving residents. Data were also collected on costs of care and potential quality-of-care indicators for facilities. Using these data, we have conducted analyses that consider several outcomes, including mortality, functional status, indicators of radiographic diagnosis of pneumonia, and costs of care. Figure 1 shows a flowchart of the project's organizational activities.

### Data Collection

All study nurses were trained with a standard protocol. To verify examination procedures, portions of evaluations were performed by different nurses (with the resident's permission) and compared immediately following the second evaluation. Additionally, the principal investigator or the co-investigator overseeing the St. Louis site shadowed each study nurse to observe evaluation skills and provided immediate feedback.

Starting with creating our data forms, we employed many standard data management procedures to minimize missing and erroneous data (Table 2). For example, we designed data forms with multiple choices and check boxes whenever possible to avoid problems with interpreting handwriting, data abstractors used a specific code to indicate that items were blank and not inadvertently omitted, and the fields for continuous variables (e.g., temperature, white blood cell count) on our forms included an appropriate number of digits, decimal points (where appropriate), and clearly labeled measurement units.

**Table 2:** Data management principles.*

General
□ Carefully plan data management well ahead of data collection [2,5-8].
□ Check for problems early, while it is still possible to correct them [5,8].
□ Provide staff with appropriate training [3,8,15].
□ Provide clear lines of authority and responsibility [18].
Data collection instruments
□ Pre-test all data collection instruments [6,18].
□ Include the version number and date on each form [17].
□ Label measurement units on data collection forms [17].
□ Develop mock tables for results and fill them in with elements from data collection forms to ensure you are collecting all the variables you need [13,14].
□ Focus efforts on the variables needed for the primary analyses [5,8].
□ Develop a detailed procedural manual for data collection [8,15,18,24]; keep a log of all decisions that alter procedures.
□ Use a specific code to indicate data elements that are intentionally blank [4].
Data security, entry and cleaning
□ Double-enter and verify all data [3-6,9,16].
□ Develop a data dictionary, including allowable and in-range responses [4-6,15,18].
□ Store both paper forms and computerized data securely [15,24].
□ Back up computerized data files regularly [5], keeping offsite copies to safeguard against a system failure [4,15].
□ Thoroughly check data for missing or potentially erroneous items [1-3,5,8,9,17]. Strategies for data checking include range and consistency checks [1-3,5,8,9,17], checking for missing data [3,5,9,17], between-form consistency checks [2,5,8,9,17], comparing forms to check whether they were collected in the proper sequence, whether forms were entered more than once [16], and whether entered forms matched up with the management database [4].
□ Never obscure or destroy original data; maintain a clear audit trail of all changes to the data [2,5,8,9,15,17].

*Additional regulations that apply to data integrity and security have been enacted since the time of our study. The Health Insurance Portability and Accountability Act (HIPAA) [25] addresses the security and privacy of health data, and 21CFR Part 11 [26] specifically addresses the reliability of electronic records.

All forms were pre-tested by investigators and research assistants. This resulted in dropping some data elements that were judged too time-consuming to find in the medical records (for example, date of the latest pneumococcal vaccine, which could require searching several years of charts for some residents). Each form included the study title, the form title, space for the subject's identification number, and a footer with the version number and date. We were fortunate to have a full-time research assistant who had extensive experience with chart abstraction. She initially trained all of the other research assistants by visiting facilities and going through the abstraction forms item-by-item. Subsequently, research assistants from each site (central Missouri and St. Louis) developed a manual that captured all of this information. We used conference calls to facilitate this process. The manual included an overview of the study forms, information on requesting and examining medical records, a decision matrix on what information to record for each type of resident (e.g. enrolled vs. evaluated but not enrolled), detailed instructions for locating and abstracting each form's data elements, Current Procedural Terminology (CPT) codes to be recorded for the economic analysis, medication lists and codes, copies of each form, and common abbreviations and medical shorthand.

Initially, we did not appreciate the complexity of our data management needs. Within a few months, we defined clear rules on which personnel were responsible for each data management task and how each task was to be completed. Early in the study, 51 evaluations were selected for complete re-abstraction by another research assistant. Abstractors compared these forms to determine where differences occurred, further standardized their methods, and reconciled any errors that were made.

All computerized data were stored on a secure network that limited access to authorized individuals and required a password for entry. Paper forms were stored in locked cabinets when not in use. We used a relational database to track enrollment, follow-up evaluations, and receipt and location (e.g., at data entry) of all forms. To ensure confidentiality, each resident was assigned a study identification number that was included on all forms in lieu of personal identifiers. After data cleaning was completed, resident names and social security numbers were completely expunged from the tracking database, as required by our IRB. All files on our computer network were backed up regularly; approximately quarterly, files were copied and stored at an offsite location so they could be recreated in case of a major system failure.

We used twenty different forms for data collection. This necessitated substantial data quality control over an extended period of time, and precluded data entry by project staff. After visual inspection for legibility and completeness, forms were sent to an on-campus data entry facility in batches of manageable size as they became available. Data cleaning was a two-step process involving data entry followed by detailed examination of the data for potential errors. To reduce typographical errors, forms were double-entered and verified; after one data entry operator entered a form, a second operator entered the same form and resolved typographical differences, if necessary. To facilitate identification and processing, we printed forms on differently colored paper. We then used SAS software, Version 6.1 of the SAS System for Windows [12] to read data batches, check for errors, correct errors, and compile batches of entered data into data sets for analysis. These procedures are summarized below. Detailed descriptions of these procedures, including input statements used for data entry and management using SAS software, are available in additional file 1.

Initial screening and enrollment forms were entered in the tracking database within a week of a resident's evaluation. The tracking database checked for internal consistency (e.g., residents who met enrollment criteria were enrolled). These data were checked and corrected before follow-up assessments. The database was also used to print out lists of individuals who should receive 30- or 90-day follow-up evaluations, lists of individuals whose records were available for abstraction, and lists of missing or inconsistent data. We used weekly meetings to distribute these lists, collect incoming forms, and discuss any problems that arose. These weekly meetings provided regular discussions of problems and solutions that were critical to the data management process. We kept minutes of all meetings. In addition to weekly project meetings, staff involved with data collection regularly met with the data manager and principal investigator. We also kept a log of issues that resulted in procedural changes. One entry reads, "If we don't know whether a medication was given in capsule or tablet form, specify tablet. (10/26/95)." Batches of forms were sent to data entry approximately monthly throughout the project. The turnaround time for data entry was typically two to four weeks.

### Data Entry and Cleaning

Prior to submitting forms for data entry, forms were visually inspected for completeness and legibility. Errors found at this stage were corrected by drawing a single horizontal line through the erroneous value, printing the correct value above or next to the original, initialing and dating the change, and adding an explanatory note when appropriate. We followed the same procedure to correct data following entry, with the exception that a specific SAS command was created for each edit. The erroneous data were never obscured, thus maintaining a clear audit trail of all changes. For each study form, we developed a data dictionary that contained several elements, including the name, description, type, allowable values (range), and maximum field width for each variable. This helped data entry personnel set up a series of data entry formats that ensured output of high quality data files. Any questions about form legibility or out-of-range responses were flagged by data entry personnel for later resolution.

**Figure 2:**
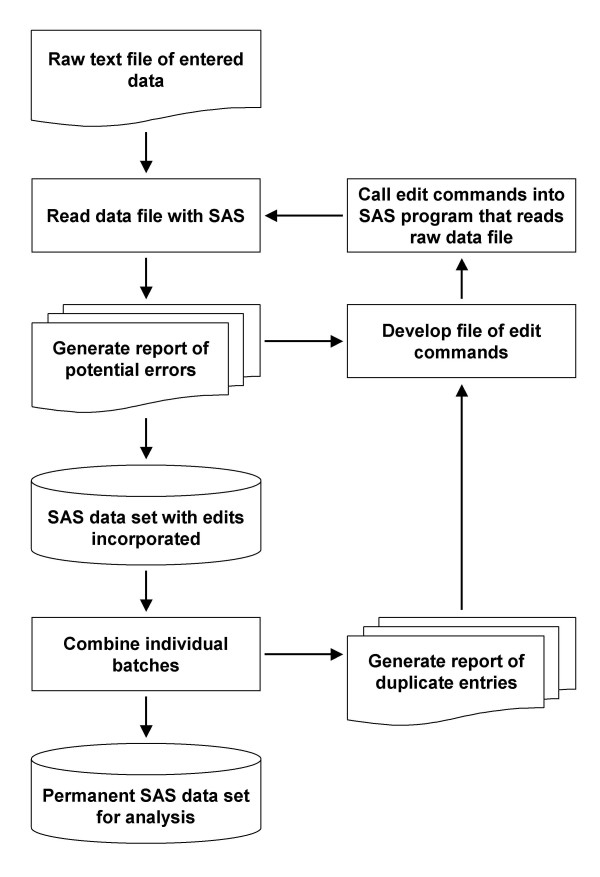
Overview of data editing process.

We established several rules to ensure accountability and data quality. Except for data entry, completed forms never left the office. The original electronic files we received from data entry were never altered. We copied each file and worked with the copies, never the originals. Each batch was given a name that identified the type of form and included a sequential number identifying the data entry batch. For example, the raw data files for participant evaluation forms for the Columbia site were named EVALCL01.DAT through EVALCL35.DAT, as there were 35 batches of entered forms. This allowed us to use simple macro variable names to refer to the batches in our SAS software programs. Figure 2 shows a flow chart of the computational tasks.

Entered data were returned to us as flat text files. Because changes must be made at specific row and column locations, directly editing a large text file can be quite difficult, particularly when each observation extends over hundreds of columns and several rows. A change made to the wrong location may be particularly difficult to find and correct. For this reason, and also to preserve the original data, the entered data files were never altered, but used to create analyzable SAS data sets. For each batch of forms, the input program read the text file, reported potential anomalies, and created a SAS data set. Each variable was given a label that included the form and a brief description of the variable. To facilitate naming almost 2,900 variables, 2–3 characters that identified forms were often used at the beginning of variable names, and variable labels included the source form as well. For example, variables names for the evaluation form usually started with EV, while those for the 90-day (quarterly) evaluation started with Q90.

The input program was primarily devoted to statements that checked the entered data for potential errors. Our strategies for checking data quality include range and consistency checks and checking for missing data. Developing boundaries for out-of-range values required a collaborative effort between the data manager and the clinician-investigators. We focused staff efforts on the highest priority data, recognizing that some missing data would simply take too much time to recover. For example, for our main outcome measure (mortality), we performed a death certificate search for the three residents who were lost to follow-up, and had no missing data. Similarly, we placed a high priority on determining activities of daily living status and body mass index. We defined high priority items as those required for determining study eligibility (e.g., vital signs, respiratory signs and symptoms, recent change in status, age, time in facility, etc.), outcomes (e.g., mortality, ADL status, health care use for the economic analysis), and variables that were considered likely covariates or confounders based on our previous work and literature review (e.g., laboratory tests, chest x-ray results, body mass index, cognitive status, comorbid diagnoses, etc.).

All editing programs were tested to make sure they detected anomalous values and did not report in-range data as anomalous using a dummy dataset containing known errors. We also made comparisons across different forms to check whether they were collected in the proper sequence, whether variables such as date of birth and gender were consistent across multiple forms, whether forms were entered more than once, and whether forms indicated as entered in the management database matched the entered data files.

All code for editing data was stored in separate files of SAS statements for each form and batch. We could thus locate the edit commands for an individual without difficulty, and the commands were easily corrected if necessary. As these files of edit commands were changed, the original text file was re-read, the edits applied, and a new SAS data set was saved. In addition to writing on paper forms, comment statements were sometimes included in the files of edit statements to provide information on why data values were changed or added. This helped preserve the "audit trail" of edits, an important process for maintaining data integrity.

Data were checked and edited as soon as possible after entry to help ensure that information was still available. After making repeat requests for some irretrievable information, we developed a computerized database of potential problems and their resolutions. This provided further documentation of all data changes and helped avoid sending staff out repeatedly to investigate the same issues.

### Creating data sets for analysis

Once the edits for each batch were complete, we appended data from each batch to a master file. Rather than waiting until all data were entered, we created interim datasets to check for consistency across datasets, check for duplicate forms, and compare entered forms with the tracking database to see if the two sources matched. Statistical analysis highlighted more potential problems, making necessary another (abbreviated) round of checking and editing. After editing was complete, we calculated the error rate for each batch with the following formula:

$$\text{Error rate}=\frac{\text{Number of edits}}{\left(\text{Number of variables on form}\right)\times \left(\text{Number of forms in batch}\right)}$$

Similarly, we summed errors and forms over all batches to derive an error rate for each type of form.

## Results

Facility nurses reported 4,959 illness episodes; after applying evaluation and exclusion criteria (Table 1), 2,592 episodes were eligible for evaluation. Physicians excluded 56 individuals from evaluation *a priori*, and residents or family members refused evaluation for 86 illness episodes of residents who were otherwise eligible. Of the 2,592 evaluations, 1,406 LRI episodes met the study definition and were enrolled, representing a total of 1,044 individuals (due to multiple enrollments). Over the course of the study, data collection resulted in 20,500 completed forms, with a combined total of 2,899 variables. The forms were entered in 418 batches, each of which was processed as described above. Early in the study, the long text (memo) fields in the project tracking database became corrupted, necessitating re-entry of some enrollment forms. Data were regularly backed up and stored offsite after this point.

Data entry, review, and correction continued throughout the study. Approximately 2/3rds of data abstraction was completed within 4 months of evaluation, with 90% completion within 6 months. Due to the time involved in logging in forms, visually inspecting forms, waiting for sufficient forms to compose a batch, preparing data entry batches, and entering and verifying data, half of the data were entered within 6 months of evaluation, with 90% complete within 9 months. Due to regular meetings between study nurses, research assistants, the principal investigator, and the data manager, we encountered very few potential errors that could not be resolved. One facility closed after study enrollment ended, and all medical records were sent to a storage facility that we could not access. Because it was unclear at the time how and whether we could gain access to the data, we decided to accept the data on the small number of records involved "as is" (only 3 of the facility's residents were enrolled in the last quarter of the study).

Despite extensive field testing of forms, we inadvertently omitted including some variables on the initial versions provided to research assistants. This resulted in adding three short forms, two of which were concerned with economic data. For early episodes for which abstraction was already complete, research assistants had to re-access medical records to make up for these omissions. Missing data were common, particularly when nurses had several ill residents to evaluate and searching the chart for the most recent height and weight, for example, was too time consuming. Many missing items were subsequently recovered in the data cleaning process. Items on which we placed a high priority, such as activities of daily living status and body mass index, had very little missing data in the final data sets (< 1%). Laboratory results had the highest proportion of missing data (>10%) [10], because tests were not always ordered and performed. An example of an error flagged by our program is a height value of 52. Height was supposed to be measured in inches, but occasionally, as in this case, feet and inches (or even centimeters) were recorded on the form. This individual was actually 5 feet and 2 inches tall, and the value was edited accordingly. An example of a non-error that was flagged for testing is a blood urea nitrogen value of 236. The normal range is 7–25 mg/dL, and we flagged values over 75 for checking against the lab report. According to the lab report, this was the resident's actual value.

Unfortunately, we did not record the specific reason for each edit (e.g., originally missing, flagged as out of range, or flagged as being inconsistent with other variables), making it impossible to determine absolute counts of all potential error sources. We did compare the raw data to the edited data for two types of forms, and discovered that most (>75%) of edits were due to missing data that were subsequently recovered. Edits to correct data entry errors were rare (<10 total for the entire study), probably because both data entry personnel must make the exact same error for this to occur. Visual inspection of forms primarily highlighted glaring problems such as skipped pages or photocopies that were illegible or cropped.

Each form had a different error rate, varying from 0.21% to 3.6% of all fields on each form. All but two forms had error rates under 1%. The form with the highest error rate, the hospital bill abstraction form, involved the complex process of placing all of the itemized charges on each bill into the proper cost category. One person abstracted all bills, followed by an item-by-item check by a second individual. The sheer number of items on bills for long hospital stays made the process difficult and prone to different interpretations.

Comparison across batches uncovered 493 duplicate or erroneous forms that were subsequently deleted or replaced. This most commonly occurred with data that were collected at baseline, 30, and 90 days. Multiple enrollments potentially overlapped, and research assistants sometimes abstracted the wrong episode's follow-up form.

Initially, we planned to devote 20% of a full-time position to data management. This proved totally inadequate; handling forms, tracking data entry, writing SAS code, producing follow-up and error reports, and applying the appropriate edits occupied approximately 1.5 FTEs. Fortunately, we had the flexibility to adapt to the unanticipated time requirements. The most time-consuming single programming task was developing the commands that checked the data for potential errors. For a large form such as the resident evaluation, error checking statements were hundreds of lines long and could take two to three days to develop. Four full-time research assistants completed data abstraction. Querying project nurses and reviewing medical charts to resolve potential errors was also time- and labor-intensive. We did not perform time studies to determine what proportion of nursing staff time was devoted to checking data, but we feel that 10% is a reasonable estimate. Such activities must be built into the project budget to ensure successfully completing data collection.

## Discussion

Data management should be planned well in advance of data collection and continue throughout a study. We used several methods to maintain data quality for over three years of data collection in the Missouri LRI Study. Regardless of whether data collection involves a small number of paper forms, direct entry into an electronic database, a sophisticated data management program, or dozens of forms over years of data collection, common principles apply.

We thought carefully about the variables we would need for analyses, but unfortunately omitted a few, requiring research assistants to re-abstract some medical records. To avoid such re-work, we recommend developing mock tables for planned publications and filling them in with variables from the data collection instruments to make sure there are no missing data elements [13,14]. Despite regular network backups, we lost some data due to file corruption; the recommendation to back up files regularly [5] should be followed. In addition to backing up data, we recommend storing copies of data files at an offsite location to avoid problems that could arise from a major system failure [4,15].

While several sources recommend visually inspecting forms for legibility and completeness prior to data entry [4,7,16,17], we found that this was primarily useful for discovering major errors such as missing pages and poor copies. Visual inspection is likely more useful for studies with small numbers of forms. In contrast, we found that the recommendation to double-enter and verify all data [3-6,9,16] led to data files with very few typographical errors. For each study form, we gave each variable a short descriptive name, and developed a data dictionary containing several elements, including the name, description, type, allowable outcomes (range), and maximum field width for each variable [4-6,15,18]. The shared data dictionary facilitated communication, collaboration, and analysis.

Despite careful planning, training, and pre-testing, some of our data files contained missing or erroneous data. We developed SAS programs to check all entered data for missing values and potential errors. *Cody's Data Cleaning Techniques Using SAS Software *[19] contains many suggestions for developing such programs. It is important to keep in mind that unlikely values are sometimes correct [1,8], and that data cleaning programs can only check for potential errors. This process is quite time consuming, but it is crucial to the overall quality of the resultant data.

We would have preferred a rapid turnaround time [5,8] between evaluation and data entry (90% completion by 9 months), but the sheer volume of the workload and budget constraints left little room for improvement. This highlights the tendency to underestimate time requirements for data management. Hogg [20] recommends carefully estimating the time and effort required for a task, and then doubling the figure to give a more realistic estimate. However, careful planning can not anticipate all problems, and flexibility to modify procedures will be needed to minimize the impact of unexpected problems [18].

The procedures described above can be incorporated into a comprehensive data management system that, if followed, will lead to high quality data for analysis. In the Missouri LRI study, missing data were minimized, and we discovered very few inconsistencies or other data problems once analysis commenced. The first author has also applied these data management procedures to other prospective cohort studies [21,22]. A limitation is that there is no way to know what we missed. In-range values could have been incorrect but never checked. It seems unlikely, however, that such potential errors would all be in one particular direction, thus biasing analyses. Replacing paper forms with electronic, on-site data entry could be used to minimize missing data, but was not feasible at the time. Galliher and colleagues [23] compared data collection on paper forms and handheld computers. For the data forms that were returned, errors of omission were much more common with the paper forms (3.5% missing items compared with 0.4% with computerized collection). They experienced technical problems with the handheld computers, however, and reported that some were stolen or lost, leading to completely missing forms. They suggested that tablet computers or data submission to a secure web site might be less prone to these types of losses.

## Conclusion

Ensuring reliable and complete data is essential to the integrity of the study. Regardless of the system used for data collection and management, rigorous application of several key principles will help ensure high quality data and facilitate analysis and interpretation of analytic results. Careful planning for data management at the beginning of a study will facilitate smooth study operation, and help keep analysis and writing on track.

## Competing interests

The authors declare that they have no competing interests.

## Authors' contributions

RK participated in the design of the study, coordinated data collection, conceived of and applied the data management strategy, participated in data analysis and interpretation, and drafted the manuscript. DM conceived of the study, and oversaw its design, coordination, and analysis. All authors read and approved the final manuscript.

## Pre-publication history

The pre-publication history for this paper can be accessed here:



## Supplementary Material

Additional file 1 **Prospective data management detailed methods**. An additional file is provided that contains methods in more detail, including examples of SAS code and output. Click here for file
