# Supplementary material for: Data management for prospective research studies using SAS® software
Source: BMC Med Res Methodol. 2008 Sep 11;8:61. doi: 10.1186/1471-2288-8-61 (PMC2546431; doi:10.1186/1471-2288-8-61)
Supplement: Additional file 1 — Prospective data management detailed methods. An additional file is provided that contains methods in more detail, including examples of SAS code and output. [file 1471-2288-8-61-S1.pdf]

Data management for prospective research studies using SAS: Detailed methods

Robin L. Kruse, PhD[1*]
David R. Mehr, MD, MS[1]

[1]Department of Family and Community Medicine
University of Missouri-Columbia School of Medicine
Columbia, MO, U.S.A.

* Corresponding author

E-mail addresses:
RLK: kruser@health.missouri.edu
DRM: mehrd@health.missouri.edu

Detailed description of data management procedures

Data Entry and Cleaning

Each batch was given a name that identified the type of form and included a sequential number identifying the data entry batch. For example, the raw data files for participant evaluation forms were named EVAL01.DAT through EVAL35.DAT, as there were 35 batches of entered forms. This allowed us to use simple macro variable names to refer to the batches in our SAS programs. We will use a very small subset of data (EVAL02.DAT in this example) to illustrate data editing and correction below. A flow chart of the overall process is shown in Figure 1.

Entered data were returned to us as flat text files, which we converted to analyzable datasets using SAS for Windows (although we originally used Version 6.1, the procedures and files described below use Version 9.1 to provide up-to-date information for readers). A portion of one data batch is shown in Figure 2. The input program to read the data and report potential anomalies (READEVAL.SAS) is shown in Figure 3. For the sake of simplicity, it is assumed that all files are stored in the subdirectory, “C:\LRISTUDY\EVALUATE.” Note that a FOOTNOTE statement with the path name of the command file is included at the beginning of the file (next to the ❶ in the left column). This will appear on all procedure output, making it easy to locate the source code among numerous subdirectories. Including the system date in the FOOTNOTE statement allows the user to determine which output is the most current. The number of the particular data batch being processed is specified in a %LET statement (❷) at the beginning of the file. We developed formats that contain allowable responses for categorical variables on each form (❸). For this form, we used a variable named EVBATCH to store the batch number with each individual’s record. The batch can thus be determined for each observation once all of the batches have been consolidated into a single analytic dataset. Assigning the batch number to each dataset can be accomplished either with a simple RETAIN statement (❹) or by having each batch’s assigned number entered in the file at data entry and reading it into SAS.

A SAS dataset is created for each batch by reading in the flat text file (❺). The INFILE statement takes advantage of the macro variable assigned with the %LET statement above (❷). Note that two periods are needed because the first period signals the end of the variable reference &DSET and the second period is part of the actual file name (EVAL02.DAT). To prevent the input file from getting too long, variable labels (❻) are kept in a separate file (EVALABEL.SAS) and called into the data step with a %INC statement. Edit statements are also stored in separate files of SAS statements for each batch (EDEVALxx.SAS, Figure 4), and are likewise called into the data step with a %INC statement (❼). This statement also requires the use of a double period for the file name to resolve properly. Before the error-checking statements are run, there will be no edit statements. In this case, or if there are no edits necessary for a particular batch, the

edit file can consist of a comment. Note that comment statements were added to the file containing the edit statements to provide information on why data values were changed or added. This is another step in the audit trail showing how and why all changes are made to the entered data. In this example, the edit statements are included before new variables are calculated and added to the data set (❽). This means, for example, that date of birth could be edited, but not age, since age is calculated after the edit statements are included. The batch is then sorted and stored as a permanent SAS dataset (❾).

The remainder of the file is devoted to statements that check the entered data for potential errors (❿). Strategies for checking data quality include range and consistency checks [1-7], checking for missing data [2,3,6,7], and between-item consistency checks [3-7]. While it is possible to write statements that check every data item on every instrument, efforts should be focused on the highest priority data [5,6]. Variables needed to assess primary outcomes should take precedence over those needed for secondary analyses. Developing boundaries for out-of-range values requires a collaborative effort of data management personnel and investigators with substantive expertise. This process can be quite time consuming but it is crucial to the overall quality of the resultant data.

Internal consistency and range checks can also be accomplished with repeated PRINT procedures, using the WHERE statement to select a particular set of conditions. The advantage of using a DATA _NULL_ step is that all potential errors for a given observation are printed out together, facilitating comparison of the printout and the paper form. Whichever method is chosen, editing programs should be tested to make sure they do detect anomalous values and that they do not report in-range data as anomalous [5,6]. The ability to detect anomalous data can be tested with a dummy dataset containing known errors. Consistency checks across forms for an individual [5] can be performed on merged datasets containing variables from two or more forms. Code can be developed to test whether forms were collected in the proper sequence, or whether variables such as date of birth, race, and gender are consistent across forms [5]. *Cody's Data Cleaning Techniques Using SAS Software* [8] contains many suggestions for developing data cleaning programs. It is important to keep in mind that unlikely values are sometimes correct [1,5], and that data cleaning programs check for potential errors.

For each batch of forms, the DATA _NULL_ step is used to generate a report with the nature of each potential error. This should be done soon after the data are received so that information is not lost [5,6]. Each query should clearly identify the participant, the data item in question, and a clear description of the problem [5-7]. Figure 5 shows the potential error report for EVAL02.DAT before including the edit statements shown in Figure 4. The output is organized by individual (❶), listing potential problems with specific variables. When potentially out-of-range values are reported (❷), the "acceptable" range and the actual value are also printed as a reference.

For small studies, simply handing this report to field personnel for investigation might be sufficient. Larger studies are better managed by maintaining a computerized database of potential problems and their resolution, including marking items as resolved or unresolvable, and the particular correction that is to be applied [1,4-7,9-11]. This provides further documentation for the audit trail of all data changes [3,4,6,7]. Keeping track of issues that can't be resolved (missing data that can't be recovered, e.g.) prevents sending study personnel out repeatedly to investigate the same items. In addition to electronic documentation, study documents should be marked manually with a single line through the erroneous value, the correct value, date, initials, and explanation [4,7].

After creating the file of editing statements to address correctable errors, the correction program should be re-run to make sure the edits were applied and that new problems weren't created. Figure 6 shows the potential error report for EVAL02.DAT after applying the edit statements shown in Figure 4. The report is shorter than the original version in Figure 5, the edits have been appropriately applied, and the remaining items are either acceptable or can't be resolved.

Creating data sets for analysis

Once the edits for a given batch are complete, the data can be appended to a master file. Figure 7 shows the program used to combine the evaluation datasets. While combining batches into a master file can wait until all edits are complete [5], creating interim datasets allows you to compare values across datasets to check for internal consistency. Combining batches also provides an opportunity to check for duplicate forms [10] and compare entered forms with the management database to see if the two sources match [9]. If interim data sets are analyzed, the date can be included in the name so that former versions are always available [4,6]. For example, the versions of the evaluation data set created in January, 1999 and July 2000 could be named EVAL9901.SAS7BDAT and EVAL0007.SAS7BDAT, respectively.

Once these final checks have been accomplished, the data file should be ready for statistical analysis. It is possible that more potential problems will be highlighted once analysis begins. Every new analysis holds the potential for uncovering new data problems. A strategy for dealing with this should be developed *a priori*. In some studies the final data sets are "locked," and no further changes are allowed. We chose to apply a final set of edits and recreate the analytic data set. At some point, however, further changes don't affect the results importantly while costing a great deal of time and effort.

References

1. Chilvers CE, Fayers PM, Freedman LS, Greenwood RM, Machin D, Palmer N, Westlake AJ: **Improving the quality of data in randomized clinical trials: the COMPACT computer package. COMPACT Steering Committee.** *Stat Med* 1988, **7:**1165-1170.

2. Tai BC, Seldrup J: **A review of software for data management, design and analysis of clinical trials.** *Ann Acad Med Singapore* 2000, **29:**576-581.

3. Hosking JD, Newhouse MM, Bagniewska A, Hawkins BS: **Data collection and transcription.** *Control Clin Trials* 1995, **16:**66S-103S.

4. Karrison T: **Data editing in a clinical trial.** *Control Clin Trials* 1981, **2:**15-29.

5. Gassman JJ, Owen WW, Kuntz TE, Martin JP, Amoroso WP: **Data quality assurance, monitoring, and reporting.** *Control Clin Trials* 1995, **16:**104S-136S.

6. Grady D, Newman TB, Vittinghoff E: **Data management.** In *Designing clinical research: an epidemiologic approach*. Edited by Hulley SB. Philadelphia, PA: Williams & Wilkins; 2001:247-257.

7. Pogash RM, Boehmer SJ, Forand PE, Dyer AM, Kunselman SJ: **Data management procedures in the Asthma Clinical Research Network.** *Control Clin Trials* 2001, **22:**168S-180S.

8. Cody RP: *Cody's data cleaning techniques using SAS software*. Cary, NC: SAS Institute Inc.; 1999.

9. DuChene AG, Hultgren DH, Neaton JD, Grambsch PV, Broste SK, Aus BM, Rasmussen WL: **Forms control and error detection procedures used at the Coordinating Center of the Multiple Risk Factor Intervention Trial (MRFIT).** *Control Clin Trials* 1986, **7:**34S-45S.

10. Pinol A, Bergel E, Chaisiri K, Diaz E, Gandeh M: **Managing data for a randomised controlled clinical trial: experience from the WHO Antenatal Care Trial. WHO Antenatal Care Trial Research Group.** *Paediatr Perinat Epidemiol* 1998, **12:**142-155.

11. Hawkins BS, Singer SW: **Design, development, and implementation of a data processing system for multiple controlled trials and epidemiologic studies.** *Control Clin Trials* 1986, **7:**89-117.

12. Tamura T, Mori H, Sugawara H: **Genome Information Broker for large and small genomes.** *Trends Genet* 1997, **13:**498.

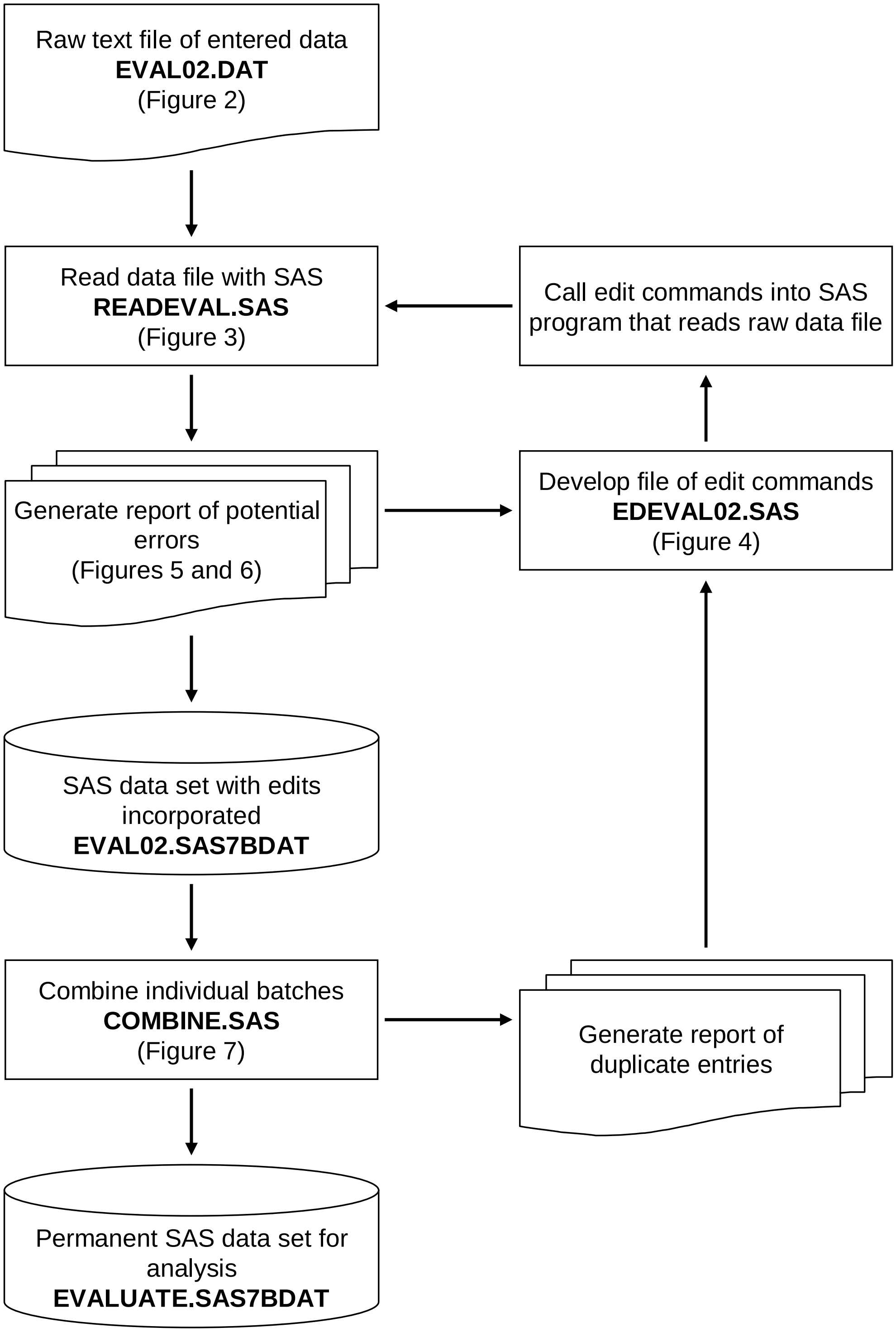

Figure 1. Overview of data editing process

```
CL1021 1        0        11221913 0 1 082995 100.2 1  92 28 138  80 59
CL1130 0 101395 1 101095 02061899 0 1 101095  36.8 1  92 28  84  47
CL1434 0 051096 1 051096 06261909 0 0 050996  97.3 1  84 44 230 130
CL1582 1        1 092896 03301900 1 1 092896 101.1 1 111 20 130  57
CL1665 0 010397 1 121396 09301910 0 1 121396 103.1 1 148 60  70  52 70 152
CL1731 1        0        08021899 0 0 122696 101.6 1  68 22 130  56 60 130 3.5
CL1669 1        0        08171922 0 1 021897  99.2 1  72 24 107  59 74 260  16
CL1711 1        0        06291922 0 0 041197 100.2 1  24 72 180  70         3.
CL1840 1        0        01121908 0 0 021997  97.8 1  60 14 110  40 62 106
CL1850 1        0        08061904 0 0 012197  99.8 1  96 28 160  80 64  77
CL1879 1        1 022297 11011907 0 0 022297 101.0 1  90 22  96  50 60 10
CL1896 1        0        10111918 0 0 021397  98.4 1  75 22 120  68 66
CL1900 1        0        10071911 0 0 021197 100.2 1 100 24 150  80 62 11
CL1934 1        0        08241911 0 0 021997 100.2 1  80 20 130  80 65 157
CL1968 1        0        07271928 0 1 031797 101.8 1  75 24 145  70 77 210 9.
CL2001 1        0        02031923 0 0 030597 100.2 1  76 20 122  84 63 145 6.4
CL2009 1        0        06121911 0 1 031397 100.4 1 100 32 110  60 69 123  12
CL2067 1                 05091906 0 0 041797 101.6 1  85 28 140  78 64 145  10
CL2130 0 042797 0        11301910 0 0 042497  99.6 1 104 28  90  64 58  87  13
CL2148 0 041397 0        02251897 0 0 040397 102.4 1  88 20         68 155 7.
CL2194 1        0        02271911 0   040797  97.6 1  80 16 150  70
CL2288 0 050997 1 050297 04291919 0 1 050297  97.4 1  72 32 110  60 73 130 8.9
CL2284 1        0        06291921 0 0 043097         72 20 120  80 64 124 7.
CL2323 0 052497 1 052197 06241928 1 1 052197  97.0 1 112 48         73 204
CL2268 1        0        07311918 0 1 050597 100.4 1  64 28 160  80 68 1
CL1989 1        0        07311922 0 0 032897                        62 125
CL2207 1        0        10161920 0 0 052397 100.2 1  80 12 108  70
CL2415 1        0        08111912 0 0 063097  99.0 1 100 20 108  60 72 138 6.
CL2466 1        0        02271923 0 1 062897  98.3 1  81 20 128  72 75 225
CL3252 1        0        10111917 0 0 121997  98.6 1  68 24 136  80 63 185 9.9
CL3294 1        0        02081920 0 1 010398 102.4 1  72 20 130  60 68 177 8.3
CL3230 1        1 122497 08241912 0 1 120997 100.4 1  80 28 140  40 68 143  16
CL1071 1        1 092595 10251927 1 1 092595  98.6 1 101 18 152  93
CL1592 1        0        11111917 0 1 100896  97.2 1 100 32 169  69         11
CL2770 1        0        04251911 0 0 100997  99.8 1  94 20 115  82 62 118  12
CL1661 0 111896 0        01191907 0         96  99.8 1  89 42 105
CL3793 1        1 04069                          0 1  68 2
```

Figure 2. Portion of flat text file of second batch of evaluation forms, EVAL02.DAT

Figure 3. SAS statements for reading batches of evaluation data

```
   *** READEVAL.SAS - READ EVALUATION DATA AND PRINT OUT DATA CHECKING REPORT ***;
   OPTIONS REPLACE;
   LIBNAME EVALDATA 'C:\LRISTUDY\EVALUATE';
   FOOTNOTE "&SYSDATE  C:\LRISTUDY\EVALUATE\READEVAL.SAS";
❶
   *** NUMBER OF DATA ENTRY BATCH TO BE PROCESSED ***;
   %LET DSET=02;
❷
   *** FORMATS WITH ALLOWABLE RESPONSES FOR DATA CHECKING ***;
   PROC FORMAT;
       VALUE VAR12MFT   .,1-2='Y'   OTHER='N';
❸      VALUE VAR0_1FT   0,1='Y'     OTHER='N';
       VALUE VAR0_2FT   0-2='Y'     OTHER='N';
       VALUE DRUGS      .,1-52,101-175,201-202,301-345,400-415='Y'  OTHER='N';
       VALUE $DRUGTYP   ' ','C','L','I','M','N','S','T','V'='Y'     OTHER='N';

   *** READ IN &DSET.DAT FILE, USING MACRO VARIABLE TO REFER TO DATA ENTRY BATCH ***;
   DATA EVALUATE (DROP=HT_IN WT_LB);
       RETAIN EVBATCH "&DSET";
       INFILE "C:\LRISTUDY\EVALUATE\EVAL&DSET..DAT" LRECL=212;
       INPUT   @1   STUDYID  $6.          @8   VITLSTAT 1.
❹              @10  DOD      MMDDYY6.     @17  HOSPTLZD 1.
❺              @19  HOSPADMT MMDDYY6.     @26  DOB      MMDDYY8.
               @35  RACE     1.           @37  SEX      1.
               @39  EVALDATE MMDDYY6.     @46  EVTEMP   5.
               @52  TEMPF_C  1.           @54  EVPULSE  3.
               @58  EVRESP   2.           @61  EVSYSTOL 3.
               @65  EVDIASTL 3.           @69  HT_IN    2.
               @72  WT_LB    3.           @76  EVWBC    3.
               @80  EVHGB    3.           @84  EVSODIUM 3.
               @88  EVPOTASS 3.           @92  EVBUN    2.
               @95  EVCREAT  3.           @99  EVALBUM  3.
               @103 EVCHOLST 3.           @107 MEDCOD1  3.
               @111 MEDBEG1  MMDDYY6.     @118 MEDEND1  MMDDYY6.
               @125 MEDTYP1  $1.          @127 MEDCOD2  3.
               @131 MEDBEG2  MMDDYY6.     @138 MEDEND2  MMDDYY6.
               @145 MEDTYP2  $1.          @147 MEDCOD3  3.
               @151 MEDBEG3  MMDDYY6.     @158 MEDEND3  MMDDYY6.
               @165 MEDTYP3  $1.          @167 MEDCOD4  3.
               @171 MEDBEG4  MMDDYY6.     @178 MEDEND4  MMDDYY6.
               @185 MEDTYP4  $1.          @187 MEDCOD5  3.
               @191 MEDBEG5  MMDDYY6.     @198 MEDEND5  MMDDYY6.
               @205 MEDTYP5  $1.          @207 ABSTDATE MMDDYY6.;
   *** INPUT DATA LABELS THAT ARE KEPT IN A SEPARATE FILE ***;
   %INC 'C:\LRISTUDY\EVALUATE\EVALABEL.SAS';
   *** INPUT DATA EDITS THAT ARE KEPT IN A SEPARATE FILE ***;
   %INC "C:\LRISTUDY\EVALUATE\EDEVAL&DSET..SAS";
   *** CONVERT HEIGHT AND WEIGHT TO METRIC AND CALCULATE BODY MASS INDEX ***;
       HT_M = (HT_IN*2.54)/100;
       WT_KG = WT_LB/2.205;
       BMI = WT_KG/(HT_M**2);
   *** CALCULATE AGE ***;
       AGE = INT(INTCK('MONTH',DOB,EVALDATE)/12);
❻      IF MONTH(DOB) = MONTH(EVALDATE) THEN AGE = AGE - (DAY(DOB) > DAY(EVALDATE));
   *** CONVERT FAHRENHEIT TEMPERATURES TO CENTIGRADE ***;
❼      IF TEMPF_C=1 THEN EVTEMP=((EVTEMP-32)*5)/9;
```

Figure 3. SAS statements for reading batches of evaluation data

```
❽ *** VARIABLE FORMATS ***;
      FORMAT MEDBEG1-MEDBEG5 MEDEND1-MEDEND5 DOB DOD EVALDATE MMDDYY8.;

  *** SAVE BATCH AS A SAS DATA SET ***;
  PROC SORT DATA=EVALUATE OUT=EVALDATA.EVAL&DSET;
      BY STUDYID;

  *** PRINT OUT REPORT OF INCONSISTENT, MISCODED & POTENTIALLY OUT OF RANGE DATA ***;
  DATA _NULL_;
      SET EVALDATA.EVAL&DSET;
      RETAIN LASTLINE 5;
      FILE PRINT HEADER=HDR NOTITLES LINE=CURRLINE N=PS;
      IF SUBSTR(STUDYID,1,2)^='CL' & SUBSTR(STUDYID,1,2)^='SL' THEN
          PUT @1 'Study ID mis-formatted' @24 STUDYID;
      IF ((EVALDATE < '01AUG1995'D & (EVALDATE^=.)) | (EVALDATE>'30SEP1998'D)) THEN
❾         PUT @1 STUDYID  @9 'Eval date out of range' @32 EVALDATE;
      IF PUT(VITLSTAT,VAR0_1FT.)='N' THEN
          PUT @1 STUDYID  @9 'Vital status mis-coded' @31 VITLSTAT;
      IF ((DOD<EVALDATE & (DOD^=.)) | (DOD>"&SYSDATE"D)) THEN
          PUT @1 STUDYID  @9 'Mismatch between eval date' @36 EVALDATE
❿             @46 'and date of death' @64 DOD;
      IF PUT(HOSPTLZD,VAR0_1FT.)='N' THEN
          PUT @1 STUDYID  @9 'Hospitalization indicator mis-coded' @45 HOSPTLZD;
      IF (HOSPTLZD=0 & HOSPADMT^=.) THEN
          PUT @1 STUDYID  @9 'Hospitalized=NO but admit date=' @41 HOSPADMT;
      IF ((HOSPTLZD=1 & HOSPADMT=.) | (HOSPADMT<EVALDATE & (HOSPADMT^=.))) THEN
          PUT @1 STUDYID  @9 'Hospitalized=YES but admit date=' @42 HOSPADMT;
      IF HOSPADMT > EVALDATE+30 THEN
          PUT @1 STUDYID  @9 'Hospital admission' @28 HOSPADMT
              @38 '> 30 days after evaluation' @65 EVALDATE;
      IF ABSTDATE < (EVALDATE + 30) THEN
          PUT @1 STUDYID  @9 'Abstraction date' @26 ABSTDATE
              @36 '< 30 days after evaluation' @63 EVALDATE;
      IF DOB=. THEN
          PUT @1 STUDYID  @9 'DOB is missing';
      IF ^(60 <= AGE < 105) & AGE^=. THEN
          PUT @1 STUDYID  @9 'Check age:' @20 AGE;
      IF PUT(RACE,VAR0_2FT.)='N' THEN
          PUT @1 STUDYID  @9 'Race incorrectly coded' @32 RACE;
      IF PUT(SEX,VAR0_1FT.)='N' THEN
          PUT @1 STUDYID  @9 'Sex incorrectly coded' @31 SEX;
      IF HT_M=. THEN
          PUT @1 STUDYID  @9 'Height is missing';
      IF ^(1.4 <= HT_M <=1.95) & HT_M^=. THEN
          PUT @1 STUDYID  @9 'Height may be out of range (1.4-1.95)' @47 HT_M 4.2;
      IF WT_KG=. THEN
          PUT @1 STUDYID  @9 'Weight is missing';
      IF ^(35 <= WT_KG <= 115) & WT_KG^=. THEN
          PUT @1 STUDYID  @9 'Weight may be out of range (35-115)' @45 WT_KG 6.2;
      IF ^(15 <= BMI <= 40) & BMI^=. THEN
          PUT @1 STUDYID  @9 'BMI may be out of range (15-40)' @41 BMI 6.2;
      IF (EVTEMP=. & EVPULSE=. & EVRESP=. & EVSYSTOL=. & EVDIASTL=.) THEN
          PUT @1 STUDYID  @9 'ALL EVALUATION VITAL SIGNS ARE BLANK';
      *** FLAG TEMPS THAT WERE INCORRECTLY CONVERTED TO CENTIGRADE ***;
      IF (1 <= EVTEMP <= 10) AND TEMPF_C=1 THEN
          PUT @1 STUDYID  @9 'Eval temp scale (F/C) miscoded';
```

Figure 3. SAS statements for reading batches of evaluation data

```
    IF ^(35 <= EVTEMP <= 40) & EVTEMP^=. THEN
        PUT @1 STUDYID  @9 'Eval temp may be out of range (35-40)' @48 EVTEMP 5.1;
    IF PUT(TEMPF_C,VAR12MFT.)='N' THEN
        PUT @1 STUDYID  @9 'Temp F/C incorrectly coded' @36 TEMPF_C;
    IF ((EVPULSE<40 & EVPULSE^=.) | (EVPULSE>140)) THEN
        PUT @1 STUDYID  @9 'Eval pulse may be out of range (40-140)' @49 EVPULSE;
    IF ((EVRESP<10 & EVRESP^=.) | (EVRESP>50)) THEN
        PUT @1 STUDYID  @9 'Eval resp rate may be out of range (10-50)' @52 EVRESP;
    IF ((EVSYSTOL<65 & EVSYSTOL^=.) | (EVSYSTOL>190)) THEN
        PUT @1 STUDYID  @9 'Eval systol BP may be out of range (65-190)' @53 EVSYSTOL;
    IF ((EVDIASTL<40 & EVDIASTL^=.) | (EVDIASTL>100)) THEN
        PUT @1 STUDYID  @9 'Eval diastol BP may be out of range (40-100)' @54 EVDIASTL;
    IF ((EVWBC<3 & EVWBC^=.) | (EVWBC>75)) THEN
        PUT @1 STUDYID  @9 'Eval WBC may be out of range (3-75)' @45 EVWBC;
    IF ((EVHGB<7 & EVHGB^=.) | (EVHGB>17)) THEN
        PUT @1 STUDYID  @9 'Eval HGB may be out of range (7-17)' @45 EVHGB;
    IF ((EVSODIUM<120 & EVSODIUM^=.) | (EVSODIUM>155)) THEN
        PUT @1 STUDYID  @9 'Eval sodium may be out of range (120-155)' @51 EVSODIUM;
    IF ((EVPOTASS<3 & EVPOTASS^=.) | (EVPOTASS>5.5)) THEN
        PUT @1 STUDYID  @9 'Eval potassium may be out of range (3-5.5)' @52 EVPOTASS;
    IF ((EVBUN<8 & EVBUN^=.) | (EVBUN>75)) THEN
        PUT @1 STUDYID  @9 'Eval BUN may be out of range (8-75)' @45 EVBUN;
    IF ((EVCREAT<.6 & EVCREAT^=.) | (EVCREAT>2.2)) THEN
        PUT @1 STUDYID  @9 'Eval creatinine may be out of range (.6-2.2)' @55 EVCREAT;
    IF ((EVALBUM<2 & EVALBUM^=.) | (EVALBUM>4.2)) THEN
        PUT @1 STUDYID  @9 'Eval albumin may be out of range (2-4.2)' @50 EVALBUM;
    IF ((EVCHOLST<65 & EVCHOLST^=.) | (EVCHOLST>300)) THEN
        PUT @1 STUDYID  @9 'Eval cholesterol may be out of range (65-300)' @55 EVCHOLST;
*** PROCESS MEDICATION RECORDS ***;
    ARRAY RXCOD [12] MEDCOD1-MEDCOD5;
    ARRAY RXBEG [12] MEDBEG1-MEDBEG5;
    ARRAY RXEND [12] MEDEND1-MEDEND5;
    ARRAY RXTYP [12] $ MEDTYP1-MEDTYP5;
    DO I=1 TO 5;
        IF (RXBEG{I} > RXEND{I}) & RXEND{I} ^= .
            THEN PUT @1 STUDYID  @9 'Drug' @14 I @16 'begin date' @27 RXBEG{I}
            @36 'is after end date' @54 RXEND{I};
        IF (RXBEG{I}<(EVALDATE-365) & RXBEG{I}^=.) | RXBEG{I}>(EVALDATE+30)
            THEN PUT @1 STUDYID  @9 'Check drug' @20 I @23 'begin date' @34 RXBEG{I}
            @44 'Eval date' @54 EVALDATE;
        IF (RXEND{I}<(EVALDATE-30) AND RXEND{I}^=.) THEN
            PUT @1 STUDYID  @9 'Check drug' @20 I @23 'end date' @32 RXEND{I}
                @42 'Eval date' @52 EVALDATE;
        IF RXEND{I}^=. AND RXBEG{I}=. THEN
            PUT @1 STUDYID  @9 'Drug' @14 I @17 'begin date missing, end date:'
                @47 RXEND{I};
        IF RXCOD{I}=. & RXBEG{I}^=. THEN
            PUT @1 STUDYID  @9 'Drug' @14 I @17 'code is missing';
        IF PUT(RXCOD{I},DRUGS.)='N' THEN
            PUT @1 STUDYID  @9 'Drug' @14 I @17 'code out of range' @35 RXCOD{I};
        IF PUT(RXTYP{I},$DRUGTYP.)='N' THEN
            PUT @1 STUDYID  @9 'Drug' @14 I @17 'type is mis-coded' @35 RXTYP{I};
        END;
    IF CURRLINE^=LASTLINE & CURRLINE>5 THEN PUT;
    LASTLINE=CURRLINE;
    RETURN;
```

Figure 3. SAS statements for reading batches of evaluation data

```
HDR:
    PUT @1 "Miscoded/out of range values for evaluation abstraction data eval&dset" /
        @1 "&SYSDATE  C:\LRISTUDY\EVALUATE\READEVAL.SAS" /
        @1 73*'-' /;
    RETURN;

RUN;
```

Figure 4. Edit statements for batch 2 of evaluation data

```
*** EDEVAL02.SAS - EDITING CODE FOR EVALUATION DATA SET EVAL02.DAT ***;

SELECT(STUDYID);
    WHEN ('CL1021') DO; *** BEGIN AND END YEARS SWITCHED ON FORM ***;
        MEDBEG4='29NOV1994'D;
        MEDEND4='14AUG1995'D;
        END;
    WHEN ('CL1071') DO; *** HEIGHT AND WEIGHT OBTAINED FROM CHART ***;
        HT_IN=67;
        WT_LB=179;
        END;
    WHEN ('CL1130') TEMPF_C = 2; *** FAHRENHEIT BOX CHECKED INSTEAD OF CENTIGRADE ***;
    WHEN ('CL1582') DO;
        HT_IN=64; *** HEIGHT AND WEIGHT OBTAINED FROM CHART ***;
        WT_LB=117;
        MEDCOD2 = 1; *** WRONG MEDICATION CODE USED ON FORM ***;
        MEDTYP2 = 'C'; *** MEDICATION WAS CAPSULE, NOT LIQUID ***;
        END;
    WHEN ('CL1592') DO; *** HEIGHT AND WEIGHT OBTAINED FROM CHART ***;
        HT_IN=69;
        WT_LB=164;
        END;
    WHEN ('CL1661') DO; *** HEIGHT AND WEIGHT OBTAINED FROM CHART ***;
        HT_IN=64;
        WT_LB=159;
        END;
    WHEN ('CL1711') DO; *** PULSE AND RESP RATE TRANSPOSED ON FORM ***;
        EVPULSE=72;
        EVRESP=24;
        END;
    WHEN ('CL1879') MEDEND1='04MAR1997'D; *** YEAR INCORRECT ON FORM ***:
    WHEN ('CL1989') MEDBEG4='28DEC1996'D; *** YEAR INCORRECT ON FORM ***;
    WHEN ('CL2067') HOSPTLZD=0; *** HOSPITALIZATION INDICATOR LEFT BLANK ***;
    WHEN ('CL2194') SEX=0; *** RESIDENT'S SEX LEFT OFF FORM ***;
    WHEN ('CL3230') DO; *** HEIGHT AND WEIGHT OBTAINED FROM CHART ***;
        HT_IN=68;
        WT_LB=141;
        END;
    OTHERWISE;
    END;
```

Figure 5. Output showing potential data errors reported by READEVAL.SAS without applying edit statements

```
Miscoded/out of range values for evaluation abstraction data eval02
06DEC05  ...\MY DOCUMENTS\DATA MANAGEMENT PAPER\READEVAL.SAS
-------------------------------------------------------------------------

❶ CL1021  Weight is missing
CL1021  Check drug 3  begin date 10/27/95  Eval date 08/29/95
CL1021  Drug 4 begin date 11/29/94 is after end date 08/14/94
CL1021  Check drug 4  end date 08/14/94  Eval date 08/29/95

CL1071  Height is missing
CL1071  Weight is missing

CL1130  Height is missing
CL1130  Weight is missing
CL1130  Eval temp scale (F/C) miscoded
CL1130  Eval temp may be out of range (35-40)    2.7
❷

CL1434  Height is missing
CL1434  Weight is missing
CL1434  Eval systol BP may be out of range (65-190) 230
CL1434  Eval diastol BP may be out of range (40-100) 130

CL1582  Height is missing
CL1582  Weight is missing

CL1592  Height is missing
CL1592  Weight is missing
CL1592  Eval creatinine may be out of range (.6-2.2)  2.4

CL1661  Height is missing
CL1661  Weight is missing
CL1661  Eval BUN may be out of range (8-75) 90
CL1661  Eval creatinine may be out of range (.6-2.2)  2.9

CL1665  Eval pulse may be out of range (40-140) 148
CL1665  Eval resp rate may be out of range (10-50) 60

CL1669  Weight may be out of range (35-115) 117.91

CL1711  Height is missing
CL1711  Weight is missing
CL1711  Eval pulse may be out of range (40-140) 24
CL1711  Eval resp rate may be out of range (10-50) 72

CL1850  Weight may be out of range (35-115)  34.92
CL1850  BMI may be out of range (15-40)  13.21

CL1879  Drug 1 begin date 02/27/97 is after end date 03/04/94
CL1879  Check drug 1  end date 03/04/94  Eval date 02/22/97

CL1968  Height may be out of range (1.4-1.95) 1.96

CL1989  ALL EVALUATION VITAL SIGNS ARE BLANK
CL1989  Drug 4 begin date 12/28/97 is after end date 02/09/97
CL1989  Check drug 4  begin date 12/28/97  Eval date 03/28/97
```

Figure 5. Output showing potential data errors reported by READEVAL.SAS without applying edit statements

```
CL1989  Check drug 4  end date 02/09/97  Eval date 03/28/97

CL2067  Hospitalization indicator mis-coded .
CL2067  Check drug 4  end date 01/28/97  Eval date 04/17/97
CL2067  Check drug 5  end date 01/31/97  Eval date 04/17/97

CL2194  Sex incorrectly coded .
CL2194  Height is missing
```

Figure 6. Output showing potential data errors reported by READEVAL.SAS after including edit statements

```
Miscoded/out of range values for evaluation abstraction data eval02
11NOV05  C:\LRISTUDY\EVALUATE\READEVAL.SAS
-------------------------------------------------------------------------

CL1021  Weight is missing
CL1021  Check drug 3  begin date 10/27/95  Eval date 08/29/95

CL1130  Height is missing
CL1130  Weight is missing

CL1434  Height is missing
CL1434  Weight is missing
CL1434  Eval systol BP may be out of range (65-190) 230
CL1434  Eval diastol BP may be out of range (40-100) 130

CL1592  Eval creatinine may be out of range (.6-2.2)  2.4

CL1661  Eval BUN may be out of range (8-75) 90
CL1661  Eval creatinine may be out of range (.6-2.2)  2.9

CL1665  Eval pulse may be out of range (40-140) 148
CL1665  Eval resp rate may be out of range (10-50) 60
CL1669  Weight may be out of range (35-115) 117.91

CL1711  Height is missing
CL1711  Weight is missing

CL1850  Weight may be out of range (35-115)  34.92
CL1850  BMI may be out of range (15-40)  13.21

CL1968  Height may be out of range (1.4-1.95) 1.96

CL1989  ALL EVALUATION VITAL SIGNS ARE BLANK
CL1989  Check drug 4  end date 02/09/97  Eval date 03/28/97

CL2067  Check drug 4  end date 01/28/97  Eval date 04/17/97
CL2067  Check drug 5  end date 01/31/97  Eval date 04/17/97

CL2194  Height is missing
CL2194  Weight is missing

CL2207  Height is missing
CL2207  Weight is missing

CL3230  Eval creatinine may be out of range (.6-2.2)  4.2
```

Figure 7. SAS program for combining batches of evaluation data

```
*** COMBINE.SAS - COMBINE BATCHES OF EVALUATION DATA ***;
OPTIONS REPLACE;
LIBNAME EVALDATA 'C:\LRISTUDY\EVALUATE';
FOOTNOTE "&SYSDATE  C:\LRISTUDY\EVALUATE\COMBINE.SAS";

*** COMBINE AND OUTPUT EVALUATION DATA (BATCHES 1-5 ONLY FOR BREVITY) ***;
DATA EVALUATE;
    SET EVALDATA.EVAL01
        EVALDATA.EVAL02
        EVALDATA.EVAL03
        EVALDATA.EVAL04
        EVALDATA.EVAL05;

*** SORT DATA AND SAVE PERMANENT SAS DATA SET ***;
PROC SORT DATA=EVALUATE OUT=EVALDATA.EVALUATE;
    BY STUDYID EVBATCH;

PROC CONTENTS DATA=EVALDATA.EVALUATE;
    TITLE 'CONTENTS OF EVALUATION ABSTRACTION DATA SET';

*** IDENTIFY AND PRINT LIST OF DUPLICATE OBSERVATIONS ***;
DATA DUPS;
    SET EVALDATA.EVALUATE;
    BY STUDYID;
    IF ^(FIRST.STUDYID & LAST.STUDYID);

PROC PRINT DATA=DUPS N;
    VAR STUDYID EVBATCH;
    TITLE 'DUPLICATE STUDYIDS IN EVALUATION DATA SET';

RUN;
```
